# Energy Management Strategy for Fuel Cell Vehicles Based on Online Driving Condition Recognition Using Dual-Model Predictive Control

**DOI:** 10.3390/s24237647

**Published:** 2024-11-29

**Authors:** Fuxiang Li, Xiaolin Wang, Xucong Bao, Ziyu Wang, Ruixuan Li

**Affiliations:** College of Automation Engineering, Nanjing University of Aeronautics and Astronautics, No. 29, Jiangjun Street, Jiangning District, Nanjing 211106, China; lifuxiang@nuaa.edu.cn (F.L.); baoxc@nuaa.edu.cn (X.B.); wangziyu_1995@nuaa.edu.cn (Z.W.); 110099109x@nuaa.edu.cn (R.L.)

**Keywords:** energy management strategy, dual-model predictive control, driving condition recognition, long short-term memory, fuel cell vehicles

## Abstract

Given the urgent challenges posed by global climate change and the ongoing energy crisis, fuel cell electric vehicles (FCEVs) have emerged as a promising solution. Incorporating sophisticated energy management strategies (EMSs) into FCEVs can significantly enhance the efficiency of the complex powertrain under diverse driving conditions. In this paper, a dual-model predictive control energy management strategy based on long short-term memory (LSTM)-based driving condition recognition is proposed to enhance the economic performance of FCEVs and robustness across diverse driving conditions. Firstly, to improve the generalization capability and adaptability of the LSTM model and to enhance the accuracy of driving condition recognition, wavelet transform (WT) is introduced into both the offline training and online application of LSTM. Secondly, to enhance the real-time performance and control effectiveness of the EMS, model predictive control (MPC) and explicit model predictive control (eMPC) are established based on a unified optimization objective and constraints. Thirdly, a dual MPC switching logic is developed using the information of driving condition prediction, ensuring the coordination of dual MPCs in practical applications and enhancing their adaptability to various conditions. Finally, an evaluation of the simulations demonstrates that the proposed dual-model predictive control energy management strategy based on wavelet transform LSTM driving condition recognition (WTL-DMPC EMS) can improve economic performance. Compared with other baselines, the energy-saving capability is remarkable, showcasing its promising performance.

## 1. Introduction

With the continuous improvement of the intelligence level of new energy vehicles, the requirements for their energy consumption performance are becoming increasingly stringent [1]. Fuel cell vehicles, due to their zero carbon emissions, are considered an ideal solution to address the energy and environmental crises [2]. However, reasonably allocating the power coupling relationship among multiple power sources poses challenges for the design of energy management strategies, especially in maintaining control effectiveness under complex and variable driving conditions [3]. Therefore, it is crucial to develop high-performance energy management strategies for FCEVs [4].

The reported EMSs are broadly categorized into three types based on their implementation: rule-based EMSs [5], optimization-based EMSs [6], and learning-based EMSs [7]. Rule-based EMSs include deterministic rule-based EMSs [8] and fuzzy rule-based EMSs [9]. Deterministic rule-based EMSs are characterized by clear and predefined rules that govern decision-making processes. These rules provide transparency and high interpretability, making it easier to understand and explain the system’s behavior. They are computationally efficient, suitable for real-time applications and embedded systems, and ensure stable system responses due to their precise control over operations. However, deterministic strategies are inherently static and struggle to adapt to complex and changing driving conditions [10]. Fuzzy rule-based EMSs employ fuzzy logic to handle incomplete or ambiguous information, offering greater adaptability. However, the performance heavily relies on the design and adjustment of fuzzy sets, making it challenging to guarantee its adaptability to varying driving conditions [11]. Optimization-based EMSs can be classified into global strategies and instantaneous strategies [12]. Global optimization-based EMSs include dynamic programming (DP) [13], Pontryagin’s minimum principle (PMP), and heuristic algorithms [14], such as the genetic algorithm (GA) [15] and particle swarm optimization algorithm (PSO) [16]. These approaches aim for theoretically optimal solutions using historical driving data but face challenges in computational complexity and are limited in their adaptability across varying driving conditions without prior information. Instantaneous optimization-based EMSs, such as equivalent consumption minimum strategy (ECMS) [17] and model predictive control (MPC) [18], demonstrate effective control performance and practical application potential. An ECMS employs an equivalent coefficient to equate electric consumption with fuel consumption, thereby minimizing instantaneous equivalent fuel consumption, but it only yields local optimal solutions [19]. However, the ECMS’s predictive capabilities are restricted as it primarily considers current power states, limiting its adaptability in practical scenarios [20]. In contrast, MPC generates optimal control inputs by forecasting state changes over prediction horizons, thereby adapting to diverse driving conditions effectively, but the real-time rolling optimization and constraints inherent in MPC’s quadratic programming create significant computational intensity, constraining its practicality [21]. To address this, the explicit model predictive control is proposed, aiming to derive state feedback control laws through multi-parameter quadratic programming for linear time-invariant (LTI) systems in eMPC [22]. The eMPC shifts online computations offline, reducing computational demands and easing microcontroller workloads. Although the control laws generated by eMPC based on LTI systems are effective, the vehicle models are highly nonlinear, and driving conditions are complex and variable, making a single control law inadequate to accommodate all operating states of the vehicle [23]. Learning-based EMSs, including reinforcement-learning [24] and heuristic-dynamic programming strategies [25], show advantages for energy savings in simulations. They rely on pre-trained models to make decisions, leveraging their generalization capabilities [26]. However, the offline learning process involves extensive data collection and complex algorithm training, which is costly and time-consuming [27]. Additionally, solving optimal control problems online imposes stringent real-time performance requirements on the controller. To sum up, eMPC balances computational complexity and real-time performance, making it a practical and effective energy management solution. However, the control law generation rules of eMPC result in less adaptability to varying driving conditions compared to MPC. Therefore, coordinating the two model predictive control algorithms has become a key focus of research.

To highlight the control effectiveness of eMPC, enhancing its adaptability is essential. Methods to improve eMPC adaptability can be summarized into speed prediction [28], driving condition recognition [29], and multi-model control [30]. Speed prediction can be divided into statistical methods [31] and learning-based methods [32]. Statistical methods utilize time-series data models and inverse prediction, predicting future speed based on past speed data. These methods are suitable for speed data with obvious periodicity and trends but cannot handle complex nonlinear relationships, limiting prediction accuracy. Learning-based methods, such as using SVM regression [33] to establish speed prediction models, have good generalization ability but high computational complexity for large datasets and are sensitive to parameter selection. Methods based on decision trees [34], random forests [35], and neural networks [36] can handle nonlinear relationships and are robust to missing values and noise, with strong adaptability. However, increased model complexity can lead to overfitting, and large training datasets bring a heavy computational burden. Driving condition recognition can be categorized into rule-based methods [37], model inference-based methods [38], and machine learning-based methods [39]. Rule-based driving condition recognition uses predefined sets of rules, judging the current driving condition based on specific conditions and logic. This method is simple, intuitive, easy to implement and adjust, and suitable for specific scenarios and clear rules, but it has poor adaptability to complex and variable driving conditions. Model inference-based methods use pre-built physical or statistical models to infer the current driving condition. These methods can perform state recognition without sensor data or when data are unreliable, having a strong theoretical basis, but model building and parameter adjustment are complex. Machine learning-based methods learn the features and patterns of driving conditions from historical data to achieve condition recognition, capable of handling complex nonlinear relationships and high-dimensional data, exhibiting excellent adaptability [40]. To give predictive control algorithms better condition adaptability, long short-term memory (LSTM)-based condition recognition methods have gradually become a research hotspot. LSTM excels at handling time-series data and capturing long-term dependencies, making it significantly advantageous in driving condition recognition [41]. Specifically, LSTM analyzes continuous time-series data such as vehicle speed, acceleration, and road types to accurately identify the current driving condition, such as urban roads, highways, and mountain roads. After identifying the driving condition, eMPC and MPC strategies can be dynamically switched according to different conditions, enabling the control system to better adapt to complex and variable driving conditions. However, LSTM lacks sensitivity to changes in the instantaneous frequency components of data, especially when dealing with non-stationary signals. Additionally, raw vehicle data typically contain noise, and using such data directly for training may affect the model’s performance [42]. Wavelet transform can effectively remove noise and extract important features [43]. Moreover, the multi-scale analysis capability provided by wavelet transform allows LSTM to simultaneously utilize features at different time scales, enhancing the response speed and accuracy to driving state changes. Multi-model control can be categorized into parallel multi-model control [44], switching multi-model control [45], and adaptive multi-model control [46]. Parallel multi-model control runs multiple eMPC controllers simultaneously, offering robustness across various system states but demanding significant computational resources due to the need for storing precomputed control policies. In contrast, switching multi-model control activates a single controller at a time, improving computational efficiency but potentially causing control discontinuities due to frequent switching. Adaptive multi-model control dynamically adjusts controller parameters in real time, enhancing stability and adaptability to changing environments. While it reduces the need for switching and ensures smoother control, it requires substantial computational power for real-time data processing and parameter tuning. In summary, combining wavelet theory with LSTM can make the model exhibit higher robustness and adaptability when facing different driving conditions and data noise.

In this context, this paper proposes a dual-model predictive control energy management strategy based on wavelet transform LSTM driving condition recognition (WTL-DMPC-EMS) to enhance the adaptability across diverse driving conditions, specially designed for FCEVs. Firstly, a dual-model predictive control algorithm is proposed, balancing the predictive capability, optimization performance, and real-time application of the energy management strategy. Secondly, a wavelet transform combined with the LSTM model is introduced to enhance the accuracy and adaptability of driving state recognition. Thirdly, a set of logic for switching dual-model predictive control based on driving state recognition is established, enhancing the adaptability of the energy management strategy to changing conditions. Finally, through the simulation of dual driving cycles, the energy-saving potential and feasibility of the proposed WTL-DMPC-EMS are evaluated.

The contributions of this paper are as follows:

1. A WTL-DMPC EMS is proposed to release the energy-saving potential of an FCEV. Switching between MPC and eMPC based on the identified vehicle-operating state optimizes energy distribution, enhancing the adaptability and real-time performance of the energy management strategy to various driving conditions.
2. A driving condition recognition method is proposed that incorporates wavelet transform into LSTM (WT-LSTM), improving feature extraction capability and model robustness.
3. A switching logic between MPC and eMPC is proposed, which enhances the matching between the algorithm and driving conditions, improving the system’s response speed.

The remainder of this paper is structured as follows. The general description of the vehicle model construction is provided in Section 2. Section 3 elaborates on the developed WTL-DMPC EMS. Section 4 discusses the simulation results and validates the superior performance of the raised strategy; this is followed by the main conclusions, which are drawn in Section 5.

## 2. Modeling

**Figure 1:**
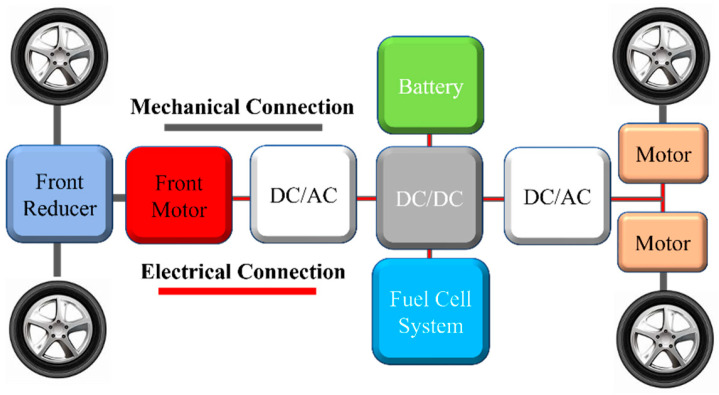
The schematic of the FCEV configuration.

The target vehicle of this paper is a fuel cell electric vehicle equipped with three motors. The vehicle configuration diagram is depicted in Figure 1. The front motor transmits torque to the front wheels through a reducer, while the two in-wheel motors directly drive the rear wheels. The specific parameters of the vehicle are demonstrated in Table 1.

### 2.1. Vehicle Dynamic

The driving force generated by the powertrain overcomes the driving resistance. At the wheel side, the dynamic characteristics of the vehicle can be expressed as

(1) $$F_{req}=\frac{mgf\cos\alpha }{3600}+\frac{mg\sin\alpha }{3600}+\frac{1}{2}\rho C_{D}Av^{2}+\frac{m\frac{dv}{dt}}{3600}$$

According to Equation (1), the vehicle power demand can be expressed as

(2) $$P_{req}=\left(\frac{mgf\cos\alpha }{3600}+\frac{mg\sin\alpha }{3600}+\frac{1}{2}\rho C_{D}Av^{2}+\frac{m\frac{dv}{dt}}{3600}\right)v$$

where m [kg] is the vehicle mass; $g\;[m/s^{2}]$ is the gravitational acceleration; α [rad] is the road slope; f is the dimensionless rolling resistance coefficient; ρ is the air density, typically taken as 1.225 $N\cdot s^{2}\cdot m^{-4}$ under standard atmospheric pressure and at a temperature of 15 °C; $C_{D}$ is the dimensionless air drag coefficient; A [$m^{2}$] is the front area of the vehicle; v [m/s] is the vehicle velocity; and $P_{req}$ [w] and $F_{req}$ [N] represent the driving force and vehicle power demand, separately. The power demand equation provided focuses solely on the longitudinal dynamics and the corresponding powertrain requirements, which are the primary factors influencing the vehicle’s energy consumption. This simplification is appropriate for the development of energy management strategies, as it directly relates to the power required to drive the vehicle forward, accelerate, and handle road slopes.

### 2.2. Fuel Cell

**Figure 2:**
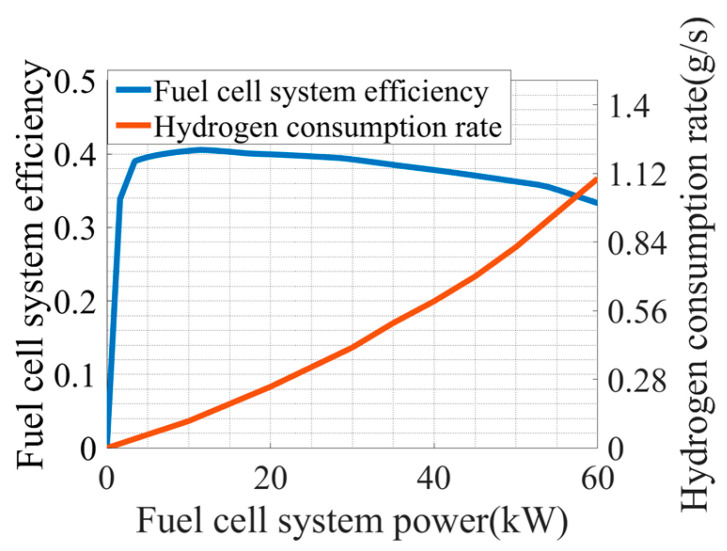
Fuel cell system efficiency and hydrogen consumption rate.

Given the focus of this paper on researching the control effect of the energy management strategy, the influence of environmental factors like temperature and humidity on the fuel cells are neglected. The relationship curves of fuel cell system efficiency, hydrogen consumption rate, and fuel cell system power are shown in Figure 2.

Theoretically, the relationship of fuel cell power $P_{FC}$ and fuel cell efficiency can be expressed as

(3) $$P_{FC}=\frac{Q_{in}}{t}\times \eta_{FC}$$

where $P_{FC}$ [w] is the fuel cell power, $\eta_{FC}$ is the fuel cell system efficiency, $Q_{in}$ [J] is the total input energy, and t is time is seconds.

The relationship between input energy and hydrogen consumption can be expressed as

(4) $$Q_{in}=m_{FC}\cdot LHV_{H_{2}}$$

(5) $$m_{FC}=\int \frac{P_{FC}}{\eta_{FC}\cdot LHV_{H_{2}}}$$

where $m_{FC}$ [kg] is the hydrogen consumption of the fuel cell and $LHV_{H_{2}}$ is the hydrogen lower heating value, here taken as 140 J/kg.

### 2.3. Battery

To improve computational efficiency, the electrochemical characteristics and temperature rise characteristics of the battery are ignored, and a simple R-C battery model is established based on experimental data. The output voltage of the battery can be expressed as

(6) $$U_{batt}=E_{batt}-I_{batt}R_{batt}$$

where $U_{batt}$ [V] is the load voltage, $E_{batt}$ [V] is the open-circuit voltage, $R_{batt}\;[\Omega ]$ is the internal resistance of the battery, and $I_{batt}$ [A] is the battery current. The definition $I_{batt}>0$ indicates charging, while $I_{batt}<0$ indicates discharging.

The state of charge (SOC) of the battery is shown as

(7) $$SOC=\left\{\begin{array}{c} SOC_{init}+\frac{1}{\eta_{batt}C_{batt}}\int_{0}^{t}\;I_{batt}dt\\ SOC_{init}-\frac{\eta_{batt}}{C_{batt}}\int_{0}^{t}\;(-I_{batt})dt \end{array}\right.$$

where $SOC_{init}$ is the dimensionless initial value of the battery’s SOC, and $C_{batt}$ is the battery capacity in Ah.

The battery efficiency $\eta_{batt}$ is defined as the ratio of battery power to total power, and it can be expressed as

(8) $$\eta_{batt}=\left\{\begin{array}{c} \frac{P_{batt}}{P_{batt}+P_{batt\_loss}}=\frac{U_{batt}I_{batt}}{U_{batt}I_{batt}+{I^{2}}_{batt}R_{batt}},\;P_{batt}<0\\ \frac{P_{batt}+P_{batt\_loss}}{P_{batt}}=\frac{U_{batt}I_{batt}+{I^{2}}_{batt}R_{batt}}{U_{batt}I_{batt}},\;P_{batt}>0 \end{array}\right.$$

where $P_{batt}$ [W] is the battery power and $P_{batt\_loss}$ [W] is the battery power loss due to internal resistance. $P_{batt}>0$ means the battery is in the charging phase. The resistance of the battery can be written as


(9) $$R_{batt}=f(SOC,temp)$$

**Figure 3:**
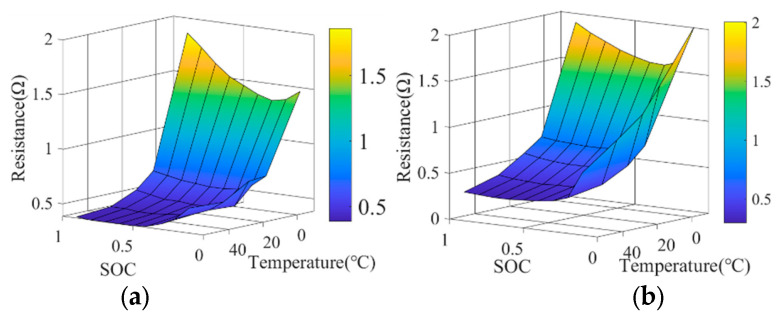
Characteristic of resistance in battery. (**a**) Resistance map during charging. (**b**) Resistance map during discharging.

The resistance characteristic of the battery during charging and discharging is shown in Figure 3.

### 2.4. Motor

As the primary power component of the target vehicle, the motor is responsible for driving and regenerative braking. To simplify the model, the thermal and dynamic features of the motor are neglected. Based on the different driving conditions of the vehicle, the power of front/rear motors can be expressed as

(10) $$P_{elec\_m}=\left\{\begin{array}{c} \frac{T_{m}\omega_{m}}{\eta_{m}},\;T_{m}>0\\ T_{m}\omega_{m}\eta_{gen},\;T_{m}\leq 0 \end{array}\right.$$

where $P_{elec\_m}$ is the electrical power of the motor, $T_{m}$ is motor torque, $\omega_{m}$ is rotating speed of the motor, $\eta_{m}$ is the efficiency of the motor during the driving mode, and $\eta_{gen}$ is the efficiency of the motor during the charging mode. $T_{m}>0$ indicates that the motor is operated in the driving mode. On the contrary, the motor is operated in the regenerative braking process.

The motor model adopts the look-up table to represent the relationship among the torque, speed, and efficiency, which can be expressed as

(11) $$\eta_{m}=\eta \left(T_{m},\omega_{m}\right)$$

**Figure 4:**
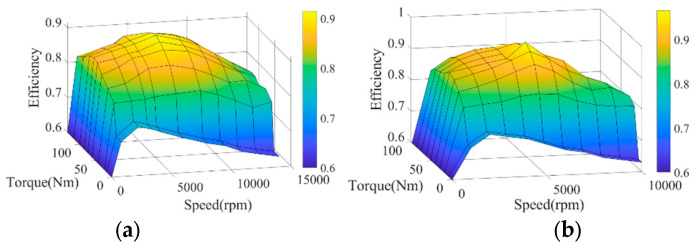
Motor efficiency map. (**a**) Efficiency map of front motor. (**b**) Efficiency map of rear motor.

The efficiency maps of the front and rear motors are provided in Figure 4.

## 3. Development of the WTL-DMPC EMS

**Figure 5:**
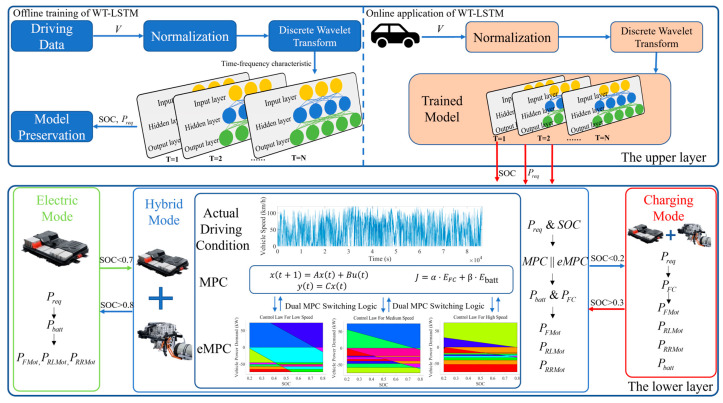
The illustration of WTL-DMPC EMS.

To equip the EMS with fast processing capacity, robustness, and practical application potential, a WTL-DMPC EMS for the studied FCEV is proposed. The general process of the EMS can be summarized as the driving condition recognition and energy management. The proposed EMS switches between MPC and eMPC by online recognition of driving conditions to solve the energy distribution problem. Figure 5 illustrates the implementation of the WTL-DMPC EMS. A hierarchical control architecture is adopted to strengthen adaptability and the control effect across diverse driving conditions. In the hierarchical control architecture, the upper layer integrates a pre-trained WT-LSTM model to recognize driving conditions in real-time and transmits the data to the lower layer. The lower layer, acting as a high-level actuator, processes the received driving condition information and switches between MPC and eMPC according to the predetermined logic to solve the energy distribution problem.

Firstly, to enhance the feature extraction and nonlinear recognition capabilities of LSTM and improve the accuracy of driving condition predictions, the wavelet transform is introduced. Secondly, to ensure the collaborative capability of the dual MPC, MPC and eMPC laws based on unified optimization objectives and constraints are established, reducing implementation complexity. Finally, to improve the adaptability of the dual MPC to various operating conditions and ensure real-time application capability, a switching logic is established, determining the priority of algorithms under different driving conditions.

### 3.1. WT-LSTM in EMS

Driving condition recognition in the upper layer of the hierarchical control architecture enables adaptive control and optimal energy management by adjusting strategies, preventing battery overcharging, and coordinating the operation of the battery fuel cell. LSTM enhances recognition accuracy by processing time-series data and capturing long-term dependencies, while wavelet transform further improves feature extraction and prediction precision.

#### 3.1.1. WT-LSTM

Long short-term memory (LSTM) networks consist of a cell state and three main gates: the forget gate, the input gate, and the output gate. These gates control the flow of information, effectively addressing the long-term dependency problem in traditional RNNs. The forget gate can be expressed as

(12) $$f_{t}=\sigma \left(W_{f}\cdot \left[h_{t-1},x_{t}\right]+b_{f}\right)$$

where $f_{t}$ is the output of the forget gate, σ is the sigmoid function, $W_{f}$ is the weight matrix for the forget gate, $\left[h_{t-1},x_{t}\right]$ is the concatenation of the previous hidden state and the current input, and $b_{f}$ is the bias vector for the forget gate.

The input gate determines which new information should be added to the cell state, which can be expressed as

(13) $$i_{t}=\sigma \left(W_{i}\cdot \left[h_{t-1},x_{t}\right]+b_{i}\right)$$

(14) $${\tilde{C}}_{t}=\tanh\left(W_{C}\cdot \left[h_{t-1},x_{t}\right]+b_{C}\right)$$

where $i_{t}$ is the output of the input gate; ${\tilde{C}}_{t}$ is the candidate cell state; $W_{i}$ and $W_{C}$ are the weight matrices for the input gate and the candidate cell state, respectively; and $b_{i}$ and $b_{C}$ are the bias vectors for the input gate and the candidate cell state, respectively.

The cell state is updated by combining the forget gate and the input gate, which can be expressed as

(15) $$C_{t}=f_{t}\cdot C_{t-1}+i_{t}\cdot {\tilde{C}}_{t}$$

where $C_{t}$ is the current cell state and $C_{t-1}$ is the previous cell state.

The output gate decides the current hidden state, which can be expressed as

(16) $$o_{t}=\sigma \left(W_{o}\cdot \left[h_{t-1},x_{t}\right]+b_{o}\right)$$

(17) $$h_{t}=o_{t}\cdot \tanh\left(C_{t}\right)$$

where $o_{t}$ is the output of the output gate, $h_{t}$ is the current hidden state, $W_{o}$ is the weight matrix for the output gate, and $b_{o}$ is the bias vector for the output gate. While LSTM excels at processing time-series data, it struggles with high-frequency and local feature extraction. To enhance its feature extraction, improve signal processing, and boost model robustness, wavelet transform is integrated into LSTM.

A wavelet function is a basic function used to generate a set of functions by scaling and translation. The wavelet function typically satisfies the following condition:

(18) $$\int_{-\infty }^{+\infty }\;\psi \left(t\right)dt=0$$

which means that the average value of the wavelet function is zero.

The scale factor a controls the dilation and compression of the wavelet function, while the translation factor b controls the position. The scaling and translation of the wavelet function produce a set of wavelet basis functions

(19) $$\psi_{a,b}\left(t\right)=\frac{1}{\sqrt{a}}\psi \left(\frac{t-b}{a}\right)$$

where $\frac{1}{\sqrt{a}}$ is a normalization factor ensuring that wavelets at different scales have the same energy.

The wavelet transform includes continuous wavelet transform (CWT) and discrete wavelet transform (DWT). The DWT has high computational efficiency and is suitable for processing large-scale data, which can be expressed as

(20) $$x\left(t\right)=\sum_{k}\;c_{j}\left[k\right]\phi_{j,k}\left(t\right)+\sum_{j}\;\sum_{k}\;d_{j}\left[k\right]\psi_{j,k}\left(t\right)$$

where $c_{j}[k]$ are the approximation coefficients, representing the signal’s low-frequency components at scale *j* and position *k*; $d_{j}[k]$ are the detail coefficients, representing the signal’s high-frequency components at scale *j* and position *k*; $\phi_{j,k}(t)$ are the scaling functions for low-frequency components; and $\psi_{j,k}(t)$ are the wavelet functions for high-frequency components.

In the offline training of WT-LSTM, the vehicle’s historical speed data are first normalized. The normalization process can be represented as

(21) $$v_{\mathrm{norm}}\left(t\right)=\frac{v\left(t\right)-v_{min}}{v_{max}-v_{min}}$$

where $v_{min}$ and $v_{max}$ are the minimum and maximum values of the speed data. Next, the normalized speed data are subjected to discrete wavelet transform, extracting multi-scale time-frequency features. The normalization in Equation (21) ensures consistent data scaling, improves model convergence, prevents bias from dominant features, enhances generalization, and stabilizes the training process.

The output of the WT-LSTM model will directly affect the accuracy of driving condition recognition and indirectly influence the control performance of MPC and eMPC. Therefore, to enhance system robustness and optimize energy distribution, SOC, vehicle speed, and vehicle power demand are chosen as the outputs of the WT-LSTM model, which can be represented as

(22) $${SOC}_{WT\text{-}LSTM}\left(t\right)=f_{SOC}\left(h_{t}\right)$$

(23) $$P_{\text{req-WT-LSTM}}\left(t\right)=f_{\mathrm{power}}\left(h_{t}\right)$$

The SOC directly affects the battery’s lifespan and performance. Accurately predicting the SOC can prevent overcharging and discharging of the battery, thus improving the overall energy efficiency of the vehicle. The vehicle power demand represents the power required under different driving conditions. Accurately predicting the power demand ensures that the vehicle has sufficient power output in all driving scenarios. Using the prediction results to dynamically switch between MPC and eMPC can optimize the overall performance of the energy management strategy. Through offline training, the WT-LSTM can be deployed in the EMS for real-time vehicle state recognition.

#### 3.1.2. The Training of WT-LSTM

The WT-LSTM model is trained offline to predict key parameters for energy management, including SOC and power demand. The process involves data preprocessing, formula derivation, and model validation, with a focus on integrating wavelet transform (WT) to enhance feature extraction. The training process uses historical vehicle speed data collected by onboard sensors. These historical data provide insights into typical driving patterns and conditions, which are essential for accurate predictions during real-time operations.

To enhance the LSTM’s ability to capture both time-domain and frequency-domain features, the wavelet transform (WT) is applied to the input data:

(24) $$X_{WT}\left(t,f\right)=\int_{-\infty }^{\infty }\;x\left(t\right)\psi^{*}\left(\frac{t-\tau }{s}\right)dt$$

where $X_{WT}(t,f)$ represents the wavelet transformed input, with s as the scaling factor and $\psi^{*}$ as the mother wavelet function. This transformation improves the LSTM’s ability to recognize transient patterns and high-frequency components in the vehicle speed data.

The LSTM cell processes the input through its hidden states:

(25) $$h_{t}=\sigma \left(W_{h}x_{t}+U_{h}h_{t-1}+b_{h}\right)$$

where $x_{t}$ is the wavelet-transformed input at time t, $h_{t-1}$ is the previous hidden state, $W_{h}$ and $U_{h}$ are weight matrices, and $b_{h}$ is the bias term.

The output predictions can be expressed as

(26) $$S\hat{O}C(t)=g\left(h_{t}\right)$$

(27) $${\hat{P}}_{\mathrm{req}\;}(t)=h\left(h_{t}\right)$$

The training process for the WT-LSTM model involves minimizing a multi-objective loss function that aims to ensure accuracy in predicting both the state of charge (SOC) and power demand of the vehicle. This comprehensive loss function balances the contributions from multiple prediction targets, making the WT-LSTM suitable for energy management in dynamic driving environments. The multi-objective loss function used in training the model can be expressed as

(28) $$L=\frac{1}{N}\sum_{i=1}^{N}\;\left(\omega {\left(SOC_{\mathrm{true},\;\;i}-S\hat{O}C_{i}\right)}^{2}+\nu {\left(P_{\mathrm{true},i}-{\hat{P}}_{\mathrm{req},\;\;i}\right)}^{2}\right)$$

where N is the number of training samples, and ω and ν are hyperparameters balancing the accuracy of the SOC and power demand predictions. The first term represents the error in the predicted SOC, while the second term represents the error in the power demand.

The combination of these loss components in a multi-objective setting allows the model to simultaneously learn multiple aspects of energy management, improving its ability to predict complex outputs under varying driving conditions. However, tuning ω and ν manually can be challenging, as different outputs may require different levels of emphasis depending on the driving scenario. To enhance the robustness of the training process and to ensure that all prediction targets are optimized effectively, a Dynamic Weight Adjustment approach is introduced.

The adaptive weighting mechanism can be implemented using a relative loss scaling approach, where the weights are updated at each training epoch:

(29) $$\omega_{t}=\frac{L_{P_{req}}}{L_{SOC}+L_{P_{req}}}$$

(30) $$\nu_{t}=\frac{L_{SOC}}{L_{SOC}+L_{P_{\mathrm{req}\;}}}$$

where $L_{SOC}$ and $L_{P_{\mathrm{req}\;}}$ represent the individual loss components for the SOC and power demand, respectively, at time step t. This approach ensures that the sum of the weights remains normalized to 1, maintaining a consistent total loss magnitude.

To further enhance the generalization ability of the WT-LSTM model, Dropout Regularization is employed during training. Dropout is a regularization technique that randomly drops a fraction of neurons from the model during each training iteration. This prevents the model from overfitting specific patterns in the training data, such as particular driving habits or conditions that are unique to a subset of historical data. In WT-LSTM, dropout is applied to both LSTM hidden states and the fully connected layer. By doing so, the model learns to distribute the representation of features across different neurons, making it less dependent on specific parts of the network and improving the model’s ability to generalize to unseen driving scenarios.

In addition to dropout, weight decay is applied to further regularize the model. The combined loss function with weight decay is represented as

(31) $$L_{\mathrm{total}\;}=L+\lambda^{*}\sum_{w\in W}\;w^{2}$$

where W represents all the weights in the network and $\lambda^{*}$ is a regularization hyperparameter controlling the strength of weight decay.

In the training process of the WT-LSTM model, a critical aspect is the use of a window size. The window size defines the number of previous time steps that the model uses to make predictions. By using a sliding window approach, the LSTM is able to capture temporal dependencies and learn the underlying patterns from historical data, which are crucial for time-series prediction tasks.

Previous work [47] and preliminary simulations indicated that incorporating a window size of 20 time steps provided an optimal balance between capturing sufficient temporal context and avoiding overfitting. This configuration allowed the LSTM to effectively learn from past data, improving prediction accuracy for key parameters such as vehicle speed, SOC, and power demand. When no window size was used, the model’s performance deteriorated, with increased prediction errors due to the inability to capture sufficient context from past data.

By incorporating these advanced training techniques, the WT-LSTM model is not only capable of accurately predicting key parameters for energy management but also demonstrates improved robustness and adaptability in complex and varying driving scenarios.

### 3.2. Energy Management Based on Dual MPC

In the upper layer of the hierarchical control architecture, the WT-LSTM uses sensors to collect real-time vehicle speed data, predict changes in the SOC and vehicle power demand, and input the predictive information into the lower layer of the hierarchical control architecture. To enhance the adaptability and real-time application capability of the energy management strategy, in the lower layer, MPC and eMPC are established to address the energy allocation problem under different operating conditions, with optimization objectives and constraints set. Considering that the eMPC law is generated based on LTI systems and that a single control law cannot meet the rapid and drastic power changes in a short period, eMPC laws suitable for different types of operating conditions are established.

#### 3.2.1. Basic MPC and eMPC

MPC uses a predictive model of the system to forecast future states and outputs. For linear systems, the predictive model is typically expressed as

(32) $$\begin{array}{l} x(t+1)=Ax(t)+Bu(t)\\ \;y(t)=Cx(t) \end{array}$$

where x(t) is the state vector of the system; u(t) is the control input vector; y(t) is the output vector; and A, B, and C are the system matrices.

The optimization objective of MPC is usually to minimize the prediction error and control input, and the objective function can be expressed as

(33) $$J=\sum_{k=0}^{N-1}\left({∥y\left(t+k|t\right)-y_{ref}\left(t+k\right)∥}_{Q}^{2}+∥u\left(t+k\right){∥}_{R}^{2}\right)+{∥y\left(t+N|t\right)-y_{ref}\left(t+N\right)∥}_{P}^{2}$$

where y(t+k∣t) is the output predicted at time t for future time t+k; $y_{ref}(t+k)$ is the reference output; Q, R, and P are weight matrices; and N is the prediction horizon length.

The control problem must respect the system constraints, including state and input constraints:

(34) $$\begin{array}{l} x_{min}\leq x(t+k|t)\leq x_{max}\\ u_{min}\leq u(t+k)\leq u_{max} \end{array}$$

where $x_{min}$ and $x_{max}$ are the minimum and maximum constraints of state vectors, respectively, and $u_{min}$ and $u_{max}$ are the minimum and maximum constraints of input vectors, respectively.

At each control interval t, the MPC solves the following optimization problem:

(35) $$\begin{array}{l} \underset{\left\{u\left(t\right),u\left(t+1\right),\ldots ,u\left(t+N-1\right)\right\}}{min}\;J\\ subject\;to:\\ x(t+k+1|t)=Ax(t+k|t)+Bu(t+k)\;for\;k=0,1,\ldots ,N-1\\ y(t+k|t)=Cx(t+k|t)\;for\;k=0,1,\ldots ,N-1\\ x_{min}\leq x(t+k|t)\leq x_{max}\;for\;k=0,1,\ldots ,N\\ u_{min}\leq u(t+k)\leq u_{max}\;for\;k=0,1,\ldots ,N-1 \end{array}$$

And then the optimal control sequence can be obtained, which can be expressed as

(36) $$\left\{u^{*}\left(t\right),u^{*}\left(t+1\right),\ldots ,u^{*}\left(t+N-1\right)\right\}$$

The first control input $u^{*}(t)$ from this sequence is applied to the following system:

(37) $$u\left(t\right)=u^{*}\left(t\right)$$

Then, the time horizon is shifted forward by one time step, and the optimization problem is solved again with updated state information at the next time step t+1. By solving this optimization problem at each time step and applying the first control input, MPC achieves optimal control performance while respecting system constraints in a receding horizon manner. However, receding horizon optimization poses challenges for the real-time application of MPC. The eMPC is an explicit solution for MPC; it introduces the multi-parameter quadratic programming to solve control problems for LTI systems. The state feedback control law generated offline is an affine relationship between the state vector and the control vector, which can avoid online rolling horizon optimization.

The problem is expressed in terms of the augmented state and control vectors:

(38) $$X=\left[\begin{array}{c} x\left(t+1|t\right)\\ x\left(t+2|t\right)\\ \vdots \\ x\left(t+N|t\right) \end{array}\right],\;U=\left[\begin{array}{c} u\left(t\right)\\ u\left(t+1\right)\\ \vdots \\ u\left(t+N-1\right) \end{array}\right]$$

Then, the system dynamics can be written in augmented form:

(39) $$X=Ax\left(t\right)+BU$$

(40) $$A=\left[\begin{array}{c} A\\ A^{2}\\ \vdots \\ A^{N} \end{array}\right],\;B=\left[\begin{array}{cccc} B & 0 & \cdots & 0\\ AB & B & \cdots & 0\\ \vdots & \ddots & \ddots & \vdots \\ A^{N-1}B & A^{N-2}B & \cdots & B \end{array}\right]$$

The cost function J can be expressed as

(41) $$J=\frac{1}{2}U^{T}HU+f^{T}U$$

where $H=B^{T}QB+R$, $f=B^{T}Q\left(Ax(t)-y_{ref}\right)$.

Convert the quadratic programming (QP) problem to a multi-parametric quadratic programming (mp-QP) problem where the state x(t) is treated as a parameter. The mp-QP problem can be formulated as

(42) $$\begin{array}{l} \underset{U}{min}\;\frac{1}{2}U^{T}HU+f(x(t){)}^{T}U\\ subject\;to:\\ GU\leq W+Sx(t) \end{array}$$

where G, W, and S are matrices derived from the constraints on x and u.

The state space is divided into polyhedral regions called critical regions (CRs). Each region corresponds to a unique active set of constraints. For each possible active set of constraints, solve the Karush–Kuhn–Tucker (KKT) conditions to find the affine control law. The KKT system can be expressed as

(43) $$\left[\begin{array}{cc} H & G_{\mathrm{active}}^{T}\\ G_{\mathrm{active}} & 0 \end{array}\right]\left[\begin{array}{c} U^{*}\\ \lambda^{*} \end{array}\right]=\left[\begin{array}{c} -f\left(x\left(t\right)\right)\\ W_{\mathrm{active}}+S_{\mathrm{active}}x\left(t\right) \end{array}\right]$$

where $G_{\mathrm{active}}$, $W_{\mathrm{active}}$, and $S_{\mathrm{active}}$ are the active sets. Solve this system to obtain

(44) $$U^{*}=K_{i}x\left(t\right)+d_{i}$$

Determine the polyhedral regions where each active set remains valid

(45) $$R_{i}=\left\{x\left(t\right)|G_{\mathrm{active}}\left(K_{i}x\left(t\right)+d_{i}\right)\leq W_{\mathrm{active}}+S_{\mathrm{active}}x\left(t\right)\right\}$$

then solve the mp-QP for all possible active sets of constraints, yielding affine control laws and corresponding critical regions. By using the above method, the eMPC state feedback control law for LTI systems can be obtained. During online application, the system state is used to identify the region, and the precomputed control law is applied for obtaining the solution. Although the initial state of the system varies at each sampling time, there is no need to repeatedly construct the optimal control problem. Instead, one can simply find the corresponding $CR_{i}$ based on the state vector, and the control vector can be obtained by the functional relationship.

Based on the foundational principles of MPC and eMPC and established FCEV model, state-space equations tailored to address the energy allocation challenge can be formulated, accompanied by the specification of an optimization objective.

#### 3.2.2. Offline Construction of Dual MPC

In the lower layer of the hierarchical control architecture, MPC and eMPC adaptively switch based on different operating conditions, enhancing the robustness and real-time performance of the EMS. Maintaining consistent state-space equations ensures that the system state does not require additional conversion or adjustment during the switching between MPC and eMPC, simplifying the switching process and reducing implementation complexity. Additionally, a unified model ensures consistency in controller design and system simulation, facilitating the validation and optimization of control strategies under different driving conditions. Therefore, during the offline computation stage, it is necessary to determine the optimization objectives and constraints, and to construct the state feedback control laws for eMPC.

To enhance the accuracy of WT-LSTM driving condition recognition, the SOC and vehicle power demand $P_{\mathrm{req}}$ are selected as the outputs of the WT-LSTM model. When switching between MPC and eMPC, maintaining consistent state variables ensures that the system state does not require additional conversion or adjustment. Furthermore, the SOC and vehicle power demand outputs from the WT-LSTM ensure that the predictive results can be promptly and accurately applied to control strategies, improving the overall performance of the system.

According to vehicle configuration, the expression of $S\dot{O}C$ can be expressed as

(46) $$S\dot{O}C=-\frac{E_{\mathrm{batt}\;}-\sqrt{E_{\mathrm{batt}\;}^{2}-4R_{\mathrm{batt}\;}P_{\mathrm{batt}\;}}}{2R_{\mathrm{batt}\;}C_{\mathrm{batt}\;}}$$

where $E_{\mathrm{batt}}$, $R_{\mathrm{batt}}$, and $C_{\mathrm{batt}\;}$ are the open-circuit voltage, internal resistance, and capacity of the battery, respectively.

The relationship between the vehicle and component power can be expressed as

(47) $$P_{\mathrm{req}}\left(t\right)=P_{batt}\left(t\right)+P_{FC}\left(t\right)$$

where $P_{batt}(t)$ is the battery power and $P_{FC}(t)$ is the fuel cell power. The power relationship with motors can be expressed as

(48) $$P_{\mathrm{batt}}\left(t\right)+P_{FC}\left(t\right)=\frac{P_{FMot}}{\eta_{FMot}}\left(t\right)+\frac{P_{RLMot}}{\eta_{RLMot}}\left(t\right)+\frac{P_{RRMot}}{\eta_{RRMot}}\left(t\right)$$

where $P_{FMot}$, $P_{RLMot}$, and $P_{RRMot}$ are the mechanical power of the front and rear motors; and $\eta_{FMot}$, $\eta_{RLMot}$, and $\eta_{RRMot}$ are the efficiency of the front and rear motors.

According to the power relationship, Equation (41) can be rewritten as

(49) $$S\dot{O}C=-\frac{E_{\mathrm{batt}\;}-\sqrt{E_{\mathrm{batt}\;}^{2}-4R_{\mathrm{batt}\;}\left(P_{\mathrm{FMot}\;}+P_{\mathrm{RLMot}\;}+P_{\mathrm{RRMot}\;}-P_{\mathrm{FC}\;}\right)}}{2R_{\mathrm{batt}\;}C_{\mathrm{batt}\;}}$$

The state matrix *A* and the input matrix *B* can be expressed as

(50) $$A=\left[\begin{array}{cc} 1 & 0\\ 0 & 1 \end{array}\right]$$

(51) $$B=\left[\begin{array}{cc} -\frac{1}{2R_{\mathrm{batt}\;C}C_{\mathrm{batt}}}\left(E_{\mathrm{batt}}-\sqrt{E_{\mathrm{batt}}^{2}-4R_{\mathrm{batt}}\left(P_{F\mathrm{Mot}}+P_{\mathrm{RLMot}}+P_{\mathrm{RRMot}}-P_{\mathrm{FC}}\right)}\right) & 0\\ 0 & 1 \end{array}\right]$$

where

(52) $$x={\left[SOC,P_{req}\right]}^{T}$$

(53) $$u={\left[P_{batt},P_{FC}\right]}^{T}$$

In establishing the optimization objective, it is necessary to consider the coordination between power sources to reduce energy consumption. The cost function can be expressed as

(54) $$J=\alpha \cdot E_{FC}+\beta \cdot E_{\mathrm{batt}\;}$$

where $E_{FC}$ is the energy consumption of the fuel cell, $E_{\mathrm{batt}\;}$ is the energy consumption of the battery, and α and β are the energy consumption weights, statically determined based on the relative costs of energy from the fuel cell and battery. In the cost function, the weights are predefined and remained constant throughout the operation. These static weights are determined based on the method proposed in previous work [48], ensuring efficient energy utilization and system durability across most operating conditions.

By adjusting the values of the two coefficients, the usage of the battery and fuel cell can be balanced. A larger α increases the weight of battery energy consumption in the cost function, prompting the controller to reduce battery usage to extend battery life. A larger β increases the weight of fuel cell energy consumption in the cost function, prompting the controller to reduce fuel cell usage to save hydrogen fuel consumption. In the WTL-DMPC EMS, the weight factors are predefined and remain constant throughout the entire operation. In this study, fixed weight factors are used, eliminating the need for real-time calculation and adjustment, thus reducing the computational burden on the control system and improving real-time performance and response speed. Additionally, fixed weights ensure consistent system response under different operating conditions, avoiding performance fluctuations due to frequent weight changes and enhancing system stability.

During the implementation of the WTL-DMPC EMS, some constraints related to the powertrain performance and vehicle dynamic should be set, which ensure that different components can operate within limits. The constraints for the optimization problem can be expressed as

(55) $$\left\{\begin{array}{l} SOC_{\min}\leq SOC(t+k)\leq SOC_{\max}\\ P_{req\_\min}\leq P_{req}(t+k)\leq P_{req\_\max}\\ P_{batt\_\min}\leq P_{batt}(t+k)\leq P_{batt\_\max}\\ P_{FC\_\min}\leq P_{FC}(t+k)\leq P_{FC\_\max}\\ P_{FMot\_\min}\leq P_{mF}\leq P_{FMot\_\max}\\ P_{RLMot\_\min}\leq P_{mRL}\leq P_{RLMot\_\max}\\ P_{RRMot\_\min}\leq P_{mRR}\leq P_{RRMot\_\max} \end{array}\right.$$

where $SOC_{\min}$ is the minimum SOC, $SOC_{\max}$ is the maximum SOC, $P_{req\_\min}$ is the minimum vehicle power demand, $P_{req\_\max}$ is the maximum vehicle power demand, $P_{batt\_\min}$ is the minimum battery power, $P_{batt\_\max}$ is the maximum battery power, $P_{FC\_\min}$ is the minimum fuel cell power, $P_{FC\_\max}$ is the maximum fuel cell power, $P_{FMot\_\min}$ is the minimum front motor power, $P_{FMot\_\max}$ is the maximum front motor power, $P_{RLMot\_\min}$ is the minimum rear left motor power, $P_{RLMot\_\max}$ is the maximum rear left motor power, $P_{RRMot\_\min}$ is the minimum rear right motor power, and $P_{RRMot\_\max}$ is the maximum rear right motor power. After determining the optimization objectives and constraints, MPC can be used to solve the energy distribution problem. However, for the eMPC, it is necessary to establish state feedback control laws offline.

The outstanding feature of the eMPC is its rapid computation capability, but it essentially solves the optimal control problem for LTI systems. During rapid changes in driving conditions, control errors can occur, necessitating its combination with MPC. To enhance the adaptability of the WTL-DMPC EMS, it is necessary to establish eMPC laws suitable for different driving conditions.

The K-means algorithm’s goal is to minimize the sum of squared distances between data points and their corresponding cluster centers. The objective function can be expressed as

(56) $$\mathrm{minimize}\sum_{j=1}^{k}\;\sum_{x_{i}\in C_{j}}\;{∥x_{i}-\mu_{j}∥}^{2}$$

where $C_{j}$ is the j-th cluster, $x_{i}$ represents a data point in cluster $C_{j}$, and $\mu_{j}$ represents the center of cluster $C_{j}$.

Randomly choose $\mu_{1},\mu_{2},\ldots ,\mu_{k}$ as initial cluster centers for each data point $x_{i}$, and then compute its distance to each cluster center and assign it to the nearest cluster:

(57) $$c_{i}=\arg\underset{j}{min}\;{∥x_{i}-\mu_{j}∥}^{2}$$

For each cluster $C_{j}$, recompute the cluster center as the mean of all data points in the cluster:

(58) $$\mu_{j}=\frac{1}{\left|C_{j}\right|}\sum_{x_{i}\in C_{j}}\;x_{i}$$

then repeat the assignment and update steps until the cluster centers do not change significantly.

By using K-means, the operating conditions are clustered into high-speed, medium-speed, and low-speed regions. $V_{Low\_1}$ and $V_{Low\_2}$ are the lower and upper limits of low-speed region, $V_{Medium\_1}$ and $V_{Medium\_2}$ are the lower and upper limits of medium-speed region, and $V_{High\_1}$ and $V_{High\_1}$ are the lower and upper limits of high-speed region.

**Figure 6:**
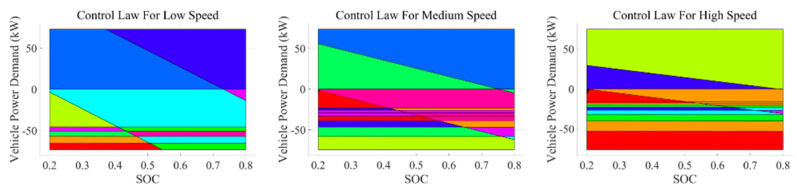
The eMPC feedback control laws.

The constant values of the eMPC’s state transition matrix A and input matrix B vary across different speed ranges, resulting in different numbers of control law regions. The established control laws are shown in Figure 6.

In the eMPC, the key regions are obtained by solving a multi-parametric quadratic programming (mp-QP) problem offline. The state space of the system is divided into several polyhedral regions, with each region corresponding to a specific set of constraints and control laws. In Figure 6, each colored region represents a particular key region, illustrating the control strategies under different states of charge (SOCs) and vehicle power demands.

The eMPC laws for different speed regions can reduce control instability caused by changes in operating conditions and improve system robustness. However, for eMPC laws, such a speed range is still quite large. Given that eMPC laws are generated based on LTI systems, it is necessary to coordinate the application of MPC and eMPC across different speed intervals.

### 3.3. Online Application of Dual MPC

To enhance the control performance and real-time capability of the WTL-DMPC EMS, and to make MPC and eMPC complementary, MPC and eMPC are switched according to different vehicle operating states in the lower layer of the hierarchical control architecture. First, the upper layer inputs the driving condition recognition information from WT-LSTM into the lower layer. Second, the lower layer dynamically switches between MPC and eMPC based on the SOC, vehicle power demand, and vehicle speed. Finally, the optimal control inputs are applied to the vehicle system.

The dual MPC is applied to solve the power distribution problem of energy sources, which requires specific problem scenarios. Typically, fuel cell vehicles operate in three driving modes: electric drive mode, hybrid mode, and driving charge mode. The hybrid mode provides a suitable working environment for the dual MPC. In hybrid mode, based on the established eMPC (extended model predictive control) control laws applicable to low-, medium-, and high-speed conditions, the switching logic between MPC and the eMPC is set.

The WT-LSTM predicts the SOC and vehicle power demand over a future period. Considering that the eMPC exhibits control errors during rapid changes in operating conditions, it is crucial to determine whether the power changes over the upcoming period are suitable for applying the eMPC. Let the vehicle power demand at the current time *t* be $P_{\mathrm{req}}(t)$, and the power predicted by WT-LSTM at time *t* + *k* be $P_{\text{req-WT-LSTM}\;}(t+k)$. The power change over the time *k* can be expressed as

(59) $$∆P_{\mathrm{req}}\left(k\right)=P_{\text{req-WT-LSTM}\;}\left(t+k\right)-P_{\mathrm{req}}\left(t\right)$$

**Figure 7:**
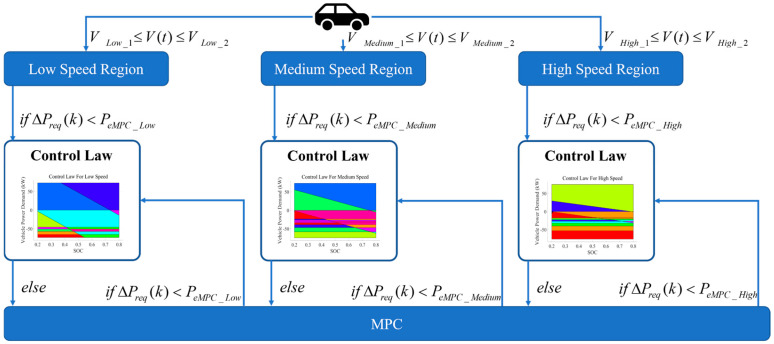
The switching logic of dual MPC.

The power change allowed by the eMPC can be denoted as $P_{\mathrm{eMPC}}$. If the power change over the time *k* exceeds the power change allowed by the eMPC, the system switches to MPC to solve the energy distribution problem. Since both MPC and the eMPC are instantaneous optimization algorithms, frequent switching is permitted. Figure 7 illustrates the switching logic between the eMPC and MPC.

As shown in Figure 7, $P_{\mathrm{eMPC}\_\mathrm{Low}\;}$ is the permitted power change in the eMPC for a low-speed region, $P_{\mathrm{eMPC}\_\mathrm{Medium}}$ is the permitted power change in the eMPC for a medium-speed region, and $P_{\mathrm{eMPC}\_\mathrm{High}}$ is the permitted power change in the eMPC for a high-speed region. Within the allowed range of power changes, the eMPC is prioritized to solve the energy distribution problem due to its excellent fast computation capabilities. Firstly, determine which speed range the current vehicle speed falls into. Secondly, assess whether the power change over time *k* is acceptable according to the control law. If acceptable, use the eMPC to solve the energy distribution problem; otherwise, use MPC to solve the power distribution problem. In operating conditions where the vehicle power demand fluctuates smoothly, the eMPC can quickly solve the optimal control problem, saving significant time compared to the online rolling optimization of MPC. However, in cases of significant changes in vehicle power demand, due to the characteristics of the eMPC’s control law, the resulting control errors are not acceptable, and MPC can provide more stable control performance.

The pattern recognition ensures that the eMPC operates in conditions that maximize its performance advantages; by classifying the current vehicle speed, the system can pre-emptively adjust the energy management strategy, ensuring seamless transitions and reducing the need for reactive control changes. By establishing the switching rules between the eMPC and MPC, the adaptability and robustness of the WTL-DMPC EMS to different operating conditions are ensured. The energy-saving performance is validated via simulation in Section 4.

## 4. Comparison of Simulation Results

After constructing the algorithm and selecting a vehicle model based on Section 2 and Section 3, respectively, the WTL-DMPC EMS for the studied FCEV is developed. This section addresses the performance evaluation of the WTL-DMPC EMS on the energy-saving validation. To evaluate the economic performance of the WTL-DMPC EMS, various driving cycles need to be tested, and the profiles are illustrated in Figures 9 and 12. The test-driving cycles are World Light Vehicle Test Cycle (WLTC) and Urban Dynamometer Driving Schedule (UDDS), representing the daily life driving conditions.

The general performance of the WTL-DMPC-EMS in optimal control is evaluated by comparing a series of baselines, including the compared energy management strategies and the matched reference curves, as follows:

- Rule-based EMS (RB): It uses a set of logic thresholds to switch the operating mode, which is benchmarked in road tests [49]. In this paper, the driving modes are divided into three categories: electric mode, hybrid mode, and charging mode. In the electric mode, the power required by the vehicle is supplied solely by the battery. In the hybrid mode, both the fuel cell and the battery provide the required power. In the charging mode, the fuel cell not only meets the vehicle’s power demand but also charges the battery. The switching logics are illustrated in Figure 6. All other baselines are built upon this foundation.
- ECMS: It is a representative instantaneous optimization-based EMS that is widely studied [50]. The equivalent factor is the core of ECMS and is used to convert battery energy into equivalent hydrogen consumption to achieve a unified measurement of different energy sources. In this paper, a fixed equivalent factor of λ = 0.55 is used, which is determined based on the average efficiency of the fuel cell and the battery. Using a fixed equivalent factor reduces the complexity of real-time calculations while ensuring a balance between energy sources.
- Model Predictive Control (MPC): It is a control algorithm with the advantages of rolling optimization, feedback correction, and is widely used in many practical engineering problems [51]. The MPC objective function balances hydrogen consumption and battery usage to ensure an optimal trade-off, as illustrated in Equation (54). The weights α is set to 0.7 and β is set to 0.3.

All of the mentioned methods have a fixed simulation step of 0.01 s. To highlight the comparison of different methods, the vehicle is in electric mode at the beginning of the simulation, and the initial SOC is set to 0.85. Simulations are performed on a computer with an Intel i5-12400 processor with 32 GB memory.

### 4.1. Assessment of Energy Consumption by Different EMSs in WLTC Driving Cycles

**Figure 8:**
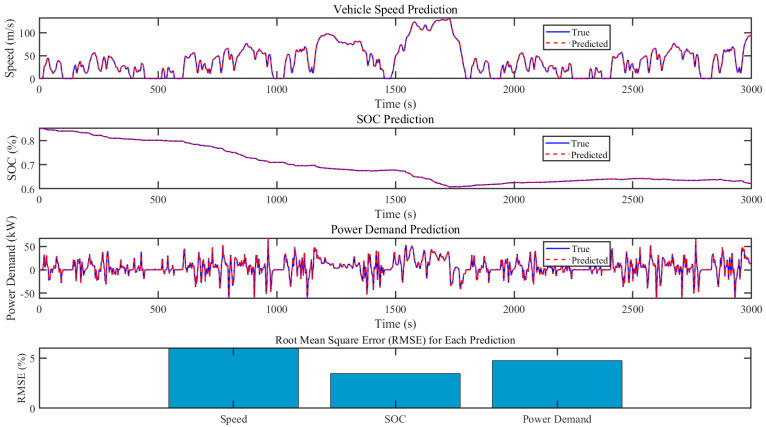
The offline training of WT-LSTM.

**Figure 9:**
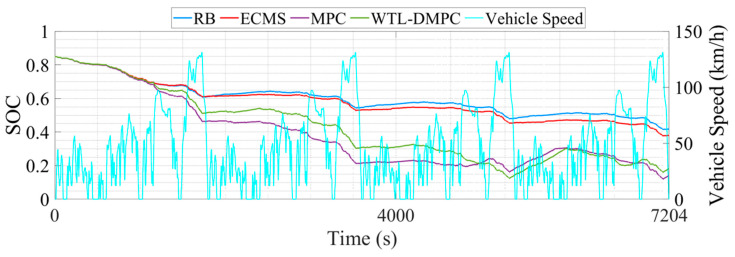
The changes in SOC in WLTC.

**Figure 10:**
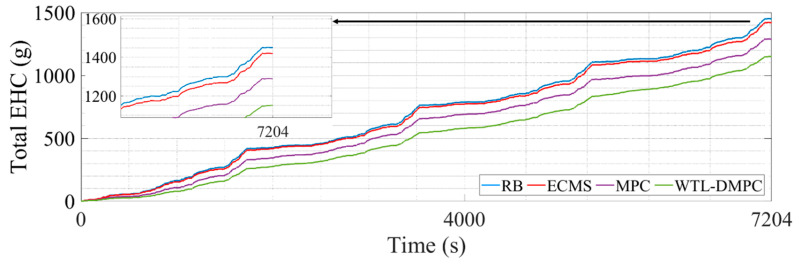
The changes in total EHC in WLTC.

This section illustrates the vehicle simulation results of the above four energy management strategies in four WLTC driving cycles. WLTC provides a comprehensive and standardized test cycle that reflects real-world driving conditions, including urban, suburban, and highway segments. This allows for a more accurate assessment of how energy management strategies perform across different driving scenarios. Moreover, WLTC includes a wide range of driving behaviors such as acceleration, deceleration, cruising, and idling. This variety ensures that energy management strategies are tested under diverse conditions, enabling a thorough evaluation of their adaptability and robustness. Table 2 lists the end SOC, the total equivalent hydrogen consumption, and the optimization percentage of total equivalent hydrogen consumption (EHC) by four EMSs. Figure 8 presents the simulation results of the WT-LSTM model. Figure 9 shows the SOC changes during the simulation. Figure 10 illustrates the changes in total equivalent hydrogen consumption. The EHC method ensures that both the fuel cell’s hydrogen consumption and the battery’s electrical energy consumption are measured on the same scale. This unified metric enables a clear comparison between the energy contributions of different power sources. The equivalent hydrogen consumption is calculated using a fixed equivalent factor to convert the battery energy into hydrogen-equivalent terms. The calculation is expressed as

(60) $$EHC=E_{batt}\times \lambda +E_{FC}$$

where EHC is the total equivalent hydrogen consumption, $E_{batt}$ is the energy used from the battery, $E_{FC}$ is the energy consumed by the fuel cell, and λ is the equivalent factor, which remains fixed throughout the calculation and reflects the equivalence between hydrogen energy and battery energy. This fixed equivalent factor λ ensures that the energy contributions from the battery are properly weighted relative to the fuel cell energy. Additionally, the weights in the objective function are predetermined and remain constant throughout the operation to reduce computational complexity. Table 2 illustrates the parameters.

Figure 8 presents the simulation results of the WT-LSTM model for predicting vehicle speed, SOC, and power demand, along with their corresponding Root Mean Square Error (RMSE) values. The first subplot illustrates the vehicle speed prediction, comparing the predicted values (red dashed line) with the true values (blue line). The close alignment of the two lines indicates a high accuracy in speed prediction over the entire 3000 s period. The second subplot depicts the SOC prediction, where the true and predicted values are almost indistinguishable, demonstrating the model’s precise performance in predicting the SOC. This high level of accuracy is further confirmed by the low RMSE value for SOC, as shown in the bar chart at the bottom of the figure. The third subplot shows the power demand prediction, with the predicted values closely tracking the true values, despite minor deviations during high-frequency fluctuations. These slight discrepancies do not significantly impact the overall prediction performance, which remains consistent and reliable. The bar chart at the bottom of the figure represents the RMSE for each predicted parameter (speed, SOC, and power demand). The relatively low RMSE values across all parameters underscore the robustness of the WT-LSTM model in capturing the complex dynamics of the vehicle and energy system. The particularly low RMSE for SOC highlights the model’s effectiveness in managing the energy state of the battery, which is critical for efficient energy management. Overall, the figure validates the accuracy and reliability of the WT-LSTM model in predicting key vehicle parameters, demonstrating its suitability for energy management strategies aimed at optimizing both fuel cell and battery operations effectively.

The RB shows a relatively stable SOC profile throughout the driving cycle. This stability suggests that RB is effective in maintaining a consistent battery charge level, ensuring that the vehicle has a reliable energy reserve. However, the stability comes at the cost of optimization. RB typically follows predefined rules without adapting to specific driving conditions, which leads to the highest total equivalent hydrogen consumption of 1450.084 g. The SOC profile for ECMS exhibits more fluctuations compared to RB. This variability is due to ECMS’s focus on minimizing equivalent fuel consumption by dynamically adjusting the power split between the fuel cell and the battery based on instantaneous efficiency calculations. While this approach can significantly reduce hydrogen consumption, it also causes more frequent charging and discharging cycles for the battery, potentially impacting its longevity. ECMS reduces hydrogen consumption, but more frequent battery activation will affect the battery lifespan. MPC further lowers the total EHC compared to ECMS and RB. By predicting future driving conditions and optimizing control actions, MPC achieves better fuel efficiency. The predictive nature of MPC allows it to make more informed decisions, reducing hydrogen consumption effectively. By judging the driving conditions in real time and switching MPC and the eMPC for energy distribution, WTL-DMPC achieves the lowest total EHC 1151.163 g among all analyzed EMSs. It combines the advantages of MPC and the eMPC, using MPC to calculate the optimal energy distribution during rapid changes in vehicle power demand, and applying eMPC laws under relatively stable conditions. Its excellent adaptability to various driving conditions enables it to demonstrate satisfactory control performance under WLTC conditions. Additionally, the WTL-DMPC has the lowest end SOC at 0.144, suggesting its aggressive optimization for fuel cell efficiency while still managing battery charge effectively. The superior performance of WTL-DMPC in reducing hydrogen consumption demonstrates its potential for real-world applications, highlighting its capability to deliver optimal fuel efficiency without compromising battery stability.

**Figure 11:**
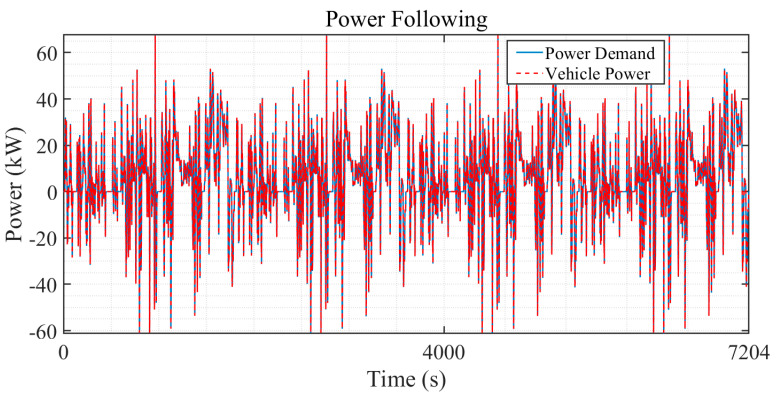
The power tracking performance of WTL-DMPC EMS under the WLTC.

Figure 11 illustrates the vehicle’s power output (red dashed line) compared to the power demand (blue solid line) over a period from 0 to 7204 s. The power demand fluctuates frequently, indicating dynamic driving conditions, and the vehicle’s power output closely follows these changes. The minimal deviation between demand and output indicates a well-performing control strategy that effectively adapts to power requirements, maintaining consistency between the powertrain response and the dynamic driving conditions.

Table 3 presents the computational time required by different EMSs during the WLTC driving cycles. Table 4 represents the energy consumption performance by different EMSs in WLTC driving cycles. The results highlight the efficiency of the WTL-DMPC strategy, which benefits from the dual MPC switching mechanism. By leveraging the fast computational capabilities of the eMPC, the simulation step time for WTL-DMPC remains within the same order of magnitude as the RB strategy, approximately 10^−4^ s. This demonstrates that the additional complexity introduced by WTL-DMPC does not lead to a significant increase in computational cost compared to RB. In contrast, both ECMS and MPC strategies require online optimization at each time step, resulting in computational costs that are an order of magnitude higher, approximately 10^−3^ s per step. The WTL-DMPC strategy, through its efficient use of dual MPC switching, showcases relatively good real-time deployment potential, combining effective energy management with computational feasibility. This makes WTL-DMPC a promising solution for practical applications requiring both high performance and computational efficiency.

The differences in strategies lead to variations in the final SOC levels. To provide a more comprehensive analysis of the WTL-DMPC performance, the SOC was restored to its initial value of 0.85 using stationary charging, and the corresponding energy consumption was converted to equivalent hydrogen consumption, as presented in Table 5. Compared to other EMSs, the proposed WTL-DMPC reveals the superior energy-saving performance. The WTL-DMPC exhibits the greatest deviation from the initial SOC, necessitating recharging to restore it to the starting level of 0.85. Despite this requirement, it achieves the lowest EHC for recharging, recorded at 1617.133 g, which is significantly lower than the consumption values of the RB, ECMS, and MPC strategies. This highlights WTL-DMPC’s effective optimization of fuel cell efficiency while managing battery utilization. Furthermore, WTL-DMPC demonstrates the highest energy-saving optimality, with a value of 6.769%, indicating its superior capability in minimizing hydrogen consumption and effectively allocating energy resources. The adaptive control and integration of both MPC and the eMPC in the proposed strategy allow for enhanced fuel efficiency under varied driving conditions.

### 4.2. Assessment of Energy Consumption by Different EMSs in UDDS Driving Cycles

**Figure 12:**
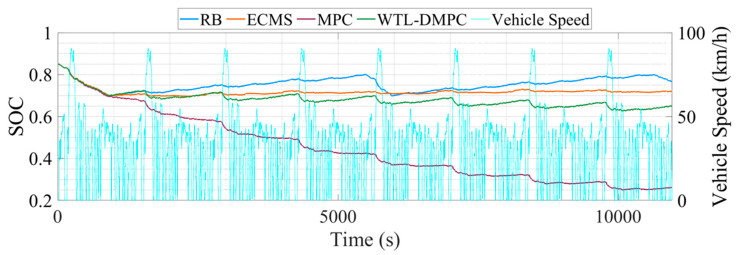
The changes in SOC in UDDS.

**Figure 13:**
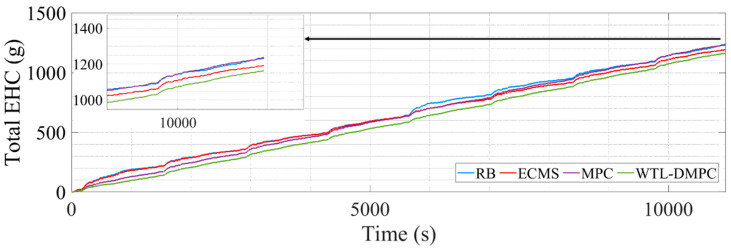
The changes in total EHC in UDDS.

This section illustrates the vehicle simulation results of the above four energy management strategies in eight UDDS driving cycles. WLTC provides a comprehensive and broad coverage of driving conditions, while UDDS offers an in-depth evaluation of urban driving details. UDDS primarily simulates city driving conditions, including frequent acceleration, deceleration, and idling phases. The dual simulation of WLTC and UDDS allows for a better assessment of the control effectiveness and robustness of energy management strategies. Table 6 lists the end SOC, the total equivalent hydrogen consumption, and the optimization percentage of total equivalent hydrogen consumption (EHC) by four EMSs. Figure 12 shows the SOC changes during the simulation. Figure 13 illustrates the changes in total equivalent hydrogen consumption.

Following the WLTC simulation, the UDDS simulation was conducted to further evaluate the performance of WTL-DMPC under urban driving conditions. In managing the SOC, Figure 12 shows that the SOC changes during the UDDS cycle indicate that WTL-DMPC continues to manage the battery charge effectively, though with some fluctuations due to the frequent start/stop nature of urban driving. Despite these fluctuations, WTL-DMPC maintains a higher SOC compared to MPC, which is crucial for ensuring sufficient energy reserves in city driving conditions. Regarding the total EHC, Figure 13 shows that WTL-DMPC achieves one of the lowest total EHC values in the UDDS simulation. This performance indicates that WTL-DMPC is efficient in managing hydrogen consumption even in urban driving conditions characterized by frequent acceleration and deceleration. As for energy-saving optimization, Table 6 reveals that WTL-DMPC achieves an energy-saving optimization of 5.972% in the UDDS simulation. While this is lower than the optimization percentage in the WLTC simulation, it still represents a significant improvement over other strategies, particularly in the context of urban driving.

WTL-DMPC shows remarkable adaptability and robustness across different driving conditions. Its ability to effectively manage SOC and minimize hydrogen consumption in both WLTC and UDDS simulations demonstrates its versatility and reliability as an energy management strategy. The dual-driving-cycle simulation approach highlights the efficacy of WTL-DMPC in both comprehensive and specific driving conditions. In WLTC, WTL-DMPC excels in the overall fuel economy, while in UDDS, it effectively handles the demands of urban driving. The excellent performance of WTL-DMPC in minimizing total EHC and maintaining SOC stability across varied driving cycles indicates that it provides optimal control performance. This makes it a promising strategy for real-world applications where vehicles encounter diverse driving scenarios.

### 4.3. Assessment of Fuel Cell Efficiency in WLTC and UDDS

**Figure 14:**
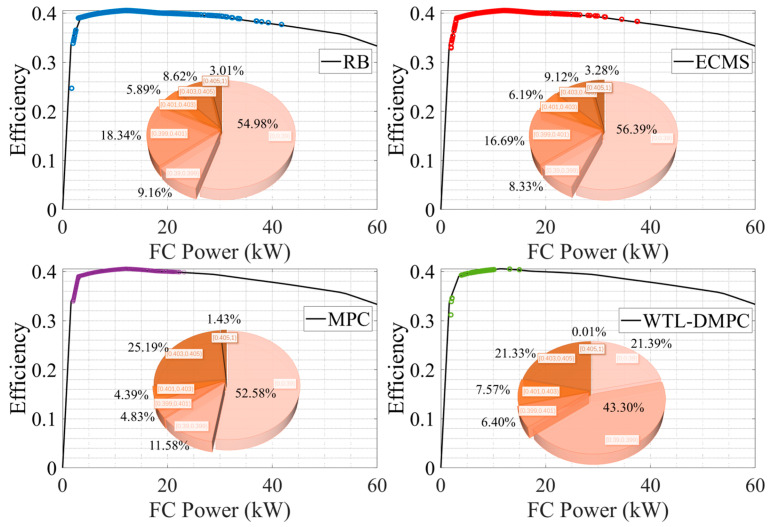
Fuel cell operating points by four EMSs in WLTC.

**Figure 15:**
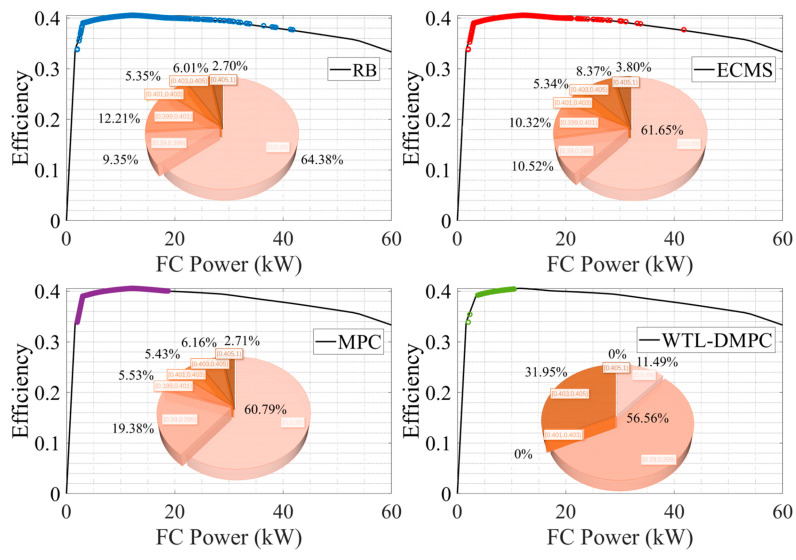
Fuel cell operating points by four EMSs in UDDS.

Figure 14 and Figure 15 show the distribution of operating points of fuel cell by different EMSs, the proportion of operating points with different efficiencies in the whole simulation process. As shown in Figure 14, in the WLTC simulation, WTL-DMPC shows a significant proportion of its fuel cell operating points in the high-efficiency range. The pie chart for WTL-DMPC indicates that 21.39% of the operating points fall into the lowest efficiency segment, which is the smallest proportion among the compared strategies. This suggests that WTL-DMPC is very effective in optimizing fuel cell operations to maximize efficiency, leading to lower hydrogen consumption and improved overall energy utilization. By maintaining a high proportion of operating points in the optimal efficiency range, WTL-DMPC ensures that the vehicle operates more economically and sustainably over a variety of driving conditions. The operating points for WTL-DMPC are well-distributed across a high-efficiency range of fuel cell power. This balanced distribution highlights WTL-DMPC’s ability to efficiently adapt to varying power demands throughout the WLTC cycle. By maintaining high efficiency across different power levels, WTL-DMPC ensures optimal performance under diverse driving conditions encountered in the WLTC. Additionally, WTL-DMPC exhibits fewer low-efficiency operating points compared to other strategies. This reduction in low-efficiency regions emphasizes WTL-DMPC’s capability to minimize energy losses and enhance fuel cell performance consistently. Avoiding low-efficiency operation contributes significantly to a more effective energy management system. By reducing the time spent in inefficient operating regions, WTL-DMPC not only conserves hydrogen but also extends the operational life of the fuel cell by preventing unnecessary stress and wear.

Under the UDDS simulation, WTL-DMPC continues to maintain a substantial proportion of high-efficiency operating points. The pie chart shows that 31.95% of the operating points are within the high-efficiency range, highlighting WTL-DMPC’s effectiveness in managing energy even in urban driving conditions characterized by frequent stops and low-speed operations. Urban driving typically involves frequent acceleration and deceleration, which can be challenging for energy management strategies. WTL-DMPC’s ability to sustain high efficiency under these conditions demonstrates its robustness and suitability for city driving. WTL-DMPC minimizes the proportion of low-efficiency operating points in the UDDS cycle. By reducing the time spent in inefficient regions, WTL-DMPC enhances overall energy management and ensures more consistent performance. This capability is particularly beneficial in urban driving, where frequent changes in speed and power demand pose challenges to less adaptive strategies. The minimization of low-efficiency operation points indicates that WTL-DMPC can handle the stop-and-go nature of urban traffic more effectively.

**Figure 16:**
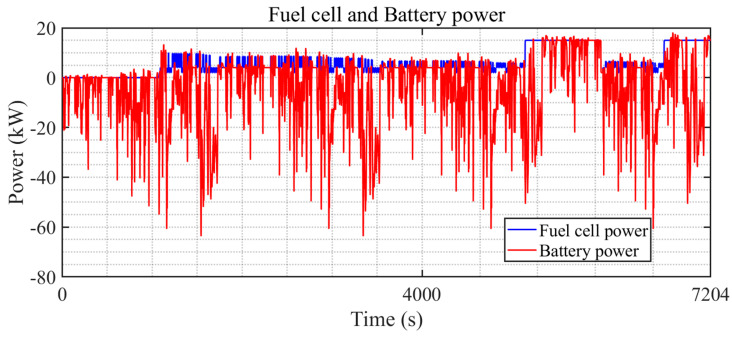
Fuel cell and battery power of WTL-DMPC in WLTC.

As illustrated in Figure 16, the battery (red line) and the fuel cell (blue line) share power demands during a driving cycle. The fuel cell maintains a relatively stable output, avoiding frequent power fluctuations, which mitigates load cycling and reduces the risk of membrane degradation and catalyst deterioration. This stability is crucial for extending the lifespan of the fuel cell. On the other hand, the battery handles the transient power demands and high-frequency fluctuations. Although the battery experiences more dynamic power variations, the WTL-DMPC strategy carefully manages the SOC within a controlled range to prevent deep discharges and overcharging. This approach helps to mitigate electrode wear and thermal stress, which are primary contributors to battery degradation. While hydrogen consumption minimization is the main optimization objective, the WTL-DMPC strategy addresses component longevity through effective power distribution. The dual MPC adaptively allocates power to minimize stress on both components, contributing to their overall durability.

In summary, WTL-DMPC stands out for its remarkable adaptability and robustness across different driving conditions, as demonstrated by the fuel cell operating points in both WLTC and UDDS simulations. The strategy’s ability to maintain high efficiency under varying power demands and driving scenarios underscores its versatility. This adaptability ensures that the vehicle can achieve optimal performance whether it is cruising on a highway or navigating through city traffic.

## 5. Conclusions

In this paper, a WTL-DMPC EMS is proposed for FCEVs, strengthening the energy-saving potential and increasing the operational efficiency of the fuel cell system. Firstly, the wavelet transform is introduced into LSTM to enhance its feature extraction capability and the accuracy of driving state prediction. Secondly, the construction of MPC and the eMPC for energy distribution problems under different driving conditions improves the adaptability and robustness of the energy management strategy, making it feasible for practical deployment. Thirdly, the proposed dual MPC switching logic increases the algorithm’s compatibility with driving states, reducing control errors during transitions in driving states. Finally, the equivalent fuel consumption of WTL-DMPC can be reduced by 20% and 6%, respectively, under dual simulation conditions.

However, aiming at guaranteeing the simulation, the required power for the studied vehicle is determined by a given a priori driving speed, which ignores the impact of predicted power error on the control effect. More effort will be devoted to the accurate required power forecasting in our future work.

## Figures and Tables

**Table 1:** Basic parameters of the studied FCEV.

**Vehicle Body**	Mass	1860 [kg]
	Tire rolling radius	0.35 [m]
**Front Motor**	Speed	0–14,000 [rpm]
	Torque	−137–137 [Nm]
**Rear Motors**	Speed	0–10,000 [rpm]
	Torque	−97–97 [Nm]
**Battery**	Nominal voltage	366 [V]
	Capacity	40 [kWh]
**Fuel Cell**	Idle Power	2 [kW]
	Maximum Power	50 [kW]

**Table 2:** The selected parameters.

Parameters	Value	Description
λ	0.55	Equivalent factor
α	0.7	Weight for fuel cell energy in the objective function
β	0.3	Weight for battery energy in the objective function
$E_{batt}$	Varies based on demand	Energy consumed from the battery
$E_{FC}$	Varies based on load	Energy consumed from the fuel cell

**Table 3:** Energy consumption performance by different EMSs in WLTC driving cycles.

EMSs	Minimum Step Time Cost (s)	Maximum Step Time Cost (s)	Average Step Time Cost (s)
RB	1.650 × 10^−4^	4.800 × 10^−4^	1.780 × 10^−4^
ECMS	1.500 × 10^−3^	1.920 × 10^−3^	1.550 × 10^−3^
MPC	1.820 × 10^−3^	2.050 × 10^−3^	1.980 × 10^−3^
WTL-DMPC	2.100 × 10^−4^	3.500 × 10^−3^	2.320 × 10^−4^

**Table 4:** Energy consumption performance by different EMSs in WLTC driving cycles.

EMSs	End SOC	Total Equivalent Hydrogen Consumption (g)	Energy-Saving Optimality (%)
RB	0.419	1450.084	-
ECMS	0.381	1419.794	2.089
MPC	0.183	1288.568	11.138
WTL-DMPC	0.144	1151.163	20.614

**Table 5:** The EHC required for stationary charging to reach the initial SOC.

EMSs	The Deviation from the Initial SOC	EHC (g)	EHC (g) for Charging to the Initial SOC	Energy-Saving Optimality (%)
RB	0.431	284.466	1734.550	-
ECMS	0.469	309.547	1729.341	0.301
MPC	0.667	440.229	1728.797	0.332
WTL-DMPC	0.706	465.970	1617.133	6.769

**Table 6:** Energy consumption performance by different EMSs in UDDS driving cycles.

EMSs	End SOC	Total Equivalent Hydrogen Consumption (g)	Energy-Saving Optimality (%)
RB	0.767	1236.592	-
ECMS	0.721	1190.864	3.698
MPC	0.262	1231.420	0.418
WTL-DMPC	0.652	1162.744	5.972

## Data Availability

The data supporting the reported results in this study are not publicly available due to privacy. Further information re-garding the data can be obtained from the corresponding author upon reasonable request.
